# Characterisation and Modelling of Moisture Gradients in Polyamide 6

**DOI:** 10.3390/polym13183141

**Published:** 2021-09-17

**Authors:** Anna Katharina Sambale, Michael Maisl, Hans-Georg Herrmann, Markus Stommel

**Affiliations:** 1 Institute of Polymer Materials, Leibniz-Institut für Polymerforschung Dresden e.V., Hohe Str. 6, D-01069 Dresden, Germany; stommel@ipfdd.de; 2 Fraunhofer Institute for Nondestructive Testing IZFP, Campus E3 1, D-66123 Saarbrücken, Germany; michael.maisl@izfp.fraunhofer.de (M.M.); hans-georg.herrmann@izfp.fraunhofer.de (H.-G.H.); 3 Chair for Lightweight Systems, Faculty of Natural Sciences and Technology, Saarland University, Campus E3 1, D-66123 Saarbrücken, Germany; 4 Chair of Polymer Materials, Institute of Materials Science, Faculty of Mechanical Science and Engineering, Technical University Dresden, D-01062 Dresden, Germany

**Keywords:** polyamide 6, water sorption, computer tomography, reconstruction method, concentration-dependent diffusion coefficients, FE modelling

## Abstract

Polyamide 6 (PA6) is able to absorb water from the surrounding air and bond to it by forming hydrogen bonds between the carbonamide groups of its molecular chains. Diffusion processes cause locally different water concentrations in the (component) cross-section during the sorption process, resulting in locally different mechanical properties due to the water-induced plasticisation effect. However, the water content of PA6 is usually specified as an integral value, so no information about a local water distribution within a component is provided. This paper shows a method to characterise moisture distributions within PA6 samples using low-energy computer tomography (CT) techniques and comparing the reconstructed results with a developed finite elements (FE) modelling method based on Fick’s diffusion laws with concentration-dependent diffusion coefficients. For this purpose, the ageing of the samples at two different water bath temperatures as well as at different integral water contents are considered. The results obtained by CT reconstruction and FE modelling are in very good agreement, so that the concentration distributions by water sorption of PA6 calculated by FEM can be regarded as validated.

## 1. Introduction

Polyamides (PAs), in particular the material PA6, have been indispensable in a multitude of technical applications for several decades: PA6 is used in particular when components are exposed to high mechanical and thermal loads in contact with different media. In technical use, however, PA6 also undergoes different climatic conditions which the material can interact with [1]: Due to its hygroscopic properties, PA6 can absorb water from its environment at higher humidities and also release it again at lower humidities until an equilibrium is reached in the material with the surrounding atmosphere. The diffusion rate of the water into the material is significantly influenced by various parameters such as temperature, load and time [2]. This leads to mainly transient conditions prevailing in the material and locally different moisture distributions [3]. The polar water molecule is attracted to the equally polar carbonamide groups within the molecular chains and attaches hydrogen bonds between the chains, so that intermolecular interactions are influenced and the intermolecular chain spacing of the material increases due to the additional space required by the diffused water molecules [2,4]. On the one hand, the water absorption results in a swelling of the material, which strongly reduces the dimensional stability of the PA components [5,6], and on the other hand, it causes the so-called water-induced plasticisation effect: The water molecules bound in the molecular chain enable the chains to slide more easily during external loads [7,8]. Water absorption thus reduces the stiffness and strength of PA6, also increases the toughness and thus considerably changes the (fracture) behaviour [5,9]. The water-induced plastification effect is also observed in the shift of the glass transition temperature $T_{g}$ from about 60 °C for dry material to about −20 °C for material completely saturated with water [5,10,11].

The water absorption of PA6 has already been characterised and modelled in various ways over the past decades. In particular, the physical mechanism of water absorption and the resulting change in stiffness of PA6 has been the focus of research then as well as now, i.e., in studies [2,4,9,12,13,14,15]. In practice, the water absorption of PA6 is mostly described by sorption curves, which represent the mass increase and thus an integral water content and therefore do not allow any conclusions regarding a local concentration distribution of the water in the material [11,16,17]. A common method for conditioning PA6 uses tempered water baths to obtain a defined integral moisture content [8]. Samples are aged in a water bath to a desired integral moisture content and then stored at defined ambient conditions such as standard climate (23 °C/50%r.H.) so that the water can be distributed evenly in the sample. Afterwards, a constant moisture content of the material in addition to a constant moisture distribution are assumed. Moisture gradients, which also involve “property gradients” due to the water-induced plasticisation, are usually not considered when characterising the material properties [8,17,18].

X-ray computed tomography (CT) provides a way to distinguish between two substances by the difference in their respective X-ray absorption. For this purpose, an energy range of the incident X-ray intensity is chosen to maximise the material-specific differences in the X-ray absorption coefficients of the substances under consideration. With the development of an appropriate reconstruction method, a penetration depth of a diffusing substance into a material can then be initially visualised and also quantified by using reference measurements. An example of such CT measurement and reconstruction method development is the work of Moradllo et al. [19], which examines the in situ ion diffusion of a potassium iodide tracer in cementitious materials using µCT, besides other methods. Sinchuk et al. [20] investigate µCT measurements to determine the diffusion of water and the induced residual stresses due to swelling processes in carbon/epoxy 3D textile composite materials with voids using the finite element method (FEM). The µCT measurements are used to generate a model in Abaqus that represents the real structure of the textile composite. Using this model, the diffusion coefficient is iteratively changed using an optimisation method until the numerically calculated sorption curves match the experimentally determined values.

In a previous work [16], an FE model was developed to numerically calculate the sorption and water-induced swelling behaviour on the basis of experimental data. Here, two consecutive analyses are performed—first, the sorption behaviour is determined using the mass diffusion analysis implemented in Abaqus (Dassault Systèmes, Vélizy-Villacoublay, France). Based on the approach of Inoue and Hoshino [21], using concentration-dependent mutual diffusion coefficients and, as suggested by Vlasveld et al. [22], considering the surface-to-volume ratio (V/A ratio) of the (sample) geometry to be calculated, the concentration distribution of water in the material is thus estimated. The calculation of the time-related percentage mass change for a computation time step is obtained from the quotient of the sum of the dissolved water quantity within the finite elements and the sum of the respective volumes of the finite elements of the FE mesh. The amount of dissolved water within an element is determined from the product of the water concentration in the finite element and the finite element volume. The results are subsequently compared with the experimentally measured sorption curves. In a second calculation step of the FE method, the temporally and locally changing concentration value is used as a field variable for a static stress analysis to determine the water-induced swelling. Here, the swelling behaviour due to water absorption is estimated on the basis of experimentally determined expansion coefficients that are direction- and concentration-dependent. Details of the FE calculation method and the experimental characterisation of the sorption and swelling behaviour of PA6 in water are shown in [16].

In the present work, the mass diffusion analysis is first applied using the concentration-dependent mutual diffusion coefficients experimentally determined in [16] to determine concentration distributions through a given sample geometry at different times of water absorption.

Furthermore, a method for measurement and reconstruction based on computed tomography (CT) is developed, which can visualise the water distribution within a sample being conditioned in a water bath and quantify the local water distribution at the respective measurement time. These measurements are used to validate the FE model of the water distribution, considering the same boundary conditions of the conditioning. Further, a possibility is shown to convert the experimentally determined concentration-dependent mutual diffusion coefficients for a variation in the water bath temperature knowing only one diffusion coefficient.

## 2. Materials and Methods

### 2.1. Sample Processing and Conditioning

For the study, bar specimens of the dimensions (4×2×80) mm³ are used. For this purpose, eight samples are produced simultaneously by an injection moulding process on an Arburg 370 S (Arburg, Loßburg, Germany) using Durethan B 31 SK (Lanxess, Cologne, Germany). The parameters of the injection moulding process are taken from the material datasheet [23]. After production, the eight samples are removed from their gating system, annealed for 100 h at 80 °C in a vacuum oven (Heraeus Holding GmbH, Hanau, Germany) and then weighed on a precision balance (HR-250A, A&D Company, Toshima, Japan) with an accuracy of 0.0001 g, so that the initial sample weight, $m_{0}$, in the dry sample state can be determined. The samples are then placed in a water bath with a defined water bath temperature (Emmi 40 HC, EMAG, Salach, Germany) for conditioning and aged until the desired integral water content is reached. The samples are then wiped dry with a lint-free cloth and weighed once more so that the mass increase can be estimated according to Equation (1). The CT measurements are performed immediately thereafter.

### 2.2. CT Measurement and Reconstruction Methods

If X-rays of intensity $I_{0}\left(E\right)\;\left[W⁄m^{2}\right]$ and energy E=h·ν [eV] iridate a material of thickness d [m] and linear X-ray absorption coefficient $\mu \;\left(E\right)\;\left[{\mathrm{cm}}^{-1}\right]$, they are attenuated to an intensity of $I\left(E\right)\;\left[W⁄m^{2}\right]$ according to the Lambert–Beer law, Equation (1).

(1) $$I\left(E\right)=I_{0}\cdot \exp\left(-\mu \cdot d\right)$$

In case of non-uniform X-ray absorption µ(x, y, z) within the object, Equation (1) is replaced by integral Equation (2).

(2) $$I_{l}=I_{0}\cdot \;\exp\cdot \;(-\int_{l}\mu \left(x,y,z\right)\mathrm{dl})$$

Reconstruction of X-ray absorption µ(x, y, z) from projections requires several projections from different directions [24,25,26]. In computer tomography, this is accomplished by rotating the object incrementally in the X-ray beam and acquiring projections by a two-dimensional X-ray detector.

The reconstruction algorithm delivers a 3D model, which is displayed as a greyscale image after a volume reconstruction. Here, each voxel is assigned to a grey scale value according to its X-ray absorption coefficient µ. The dimensions of the voxels result from the pixel height of the two-dimensional detector and the distance of the sample to the detector, and determine the resolution of the measurement [24,26].

**Figure 1:**
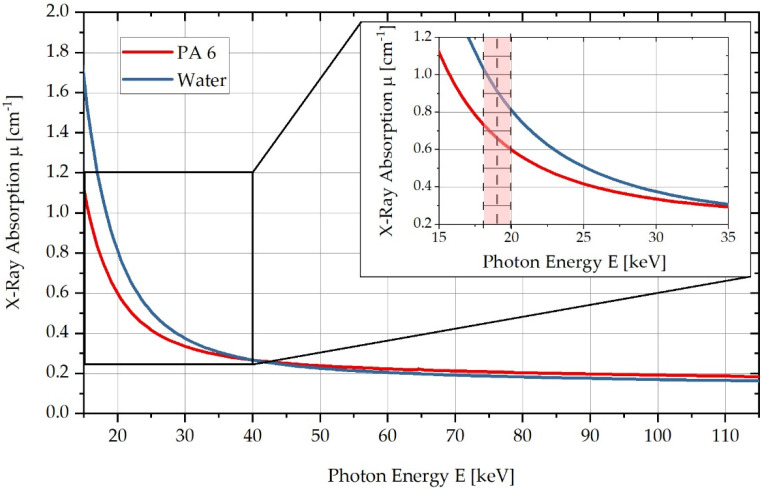
Comparison of X-ray absorption μ for PA6 and $H_{2}O$ in the energy range from 15 to 115 keV, highlighting the energy range used in a zoomed-in diagram.

PA6 and water differ in their respective linear X-ray absorption coefficients μ(E) as a function of energy. These energy-dependent absorption coefficients can be calculated using the photon cross-sections database, XCOM [27]. Figure 1 shows the respective curves of the linear X-ray absorption coefficient μ of the two substances as a function of the photon energy E [keV]. Here, it can be seen that in the red-marked energy range below 20 keV, a sufficiently large absorption difference exists to differentiate water and PA6 within this energy range in CT measurements. According to Figure 1, the X-ray absorption coefficient of PA6 increases by approx. 3.5% at a maximum water absorption of 10%.

Based on the presented absorption differences at photon energies of <20 keV, a low-energy CT system consisting of a 50 kV X-ray tube with a maximum anode current of 1 mA and a focal spot of 50 µm is set up for measurement. A 14 bit flat-panel converter with a pixel width of 200 µm and 1024×1024 elements of the type XRD 0820 is used as a detector. Within preliminary tests, the optimised measurement parameters listed in Table 1 are identified for the setup.

Due to the 1-mm-thick aluminum prefilter used, the effective X-ray voltage is roughly 19 keV. Due to a fluctuation in the high-voltage supply of the X-ray tube of approximately 600 V, there is an inaccuracy in the reconstructed absorption values of approximately ±5%, which is considered in the following reconstruction.

**Figure 2:**
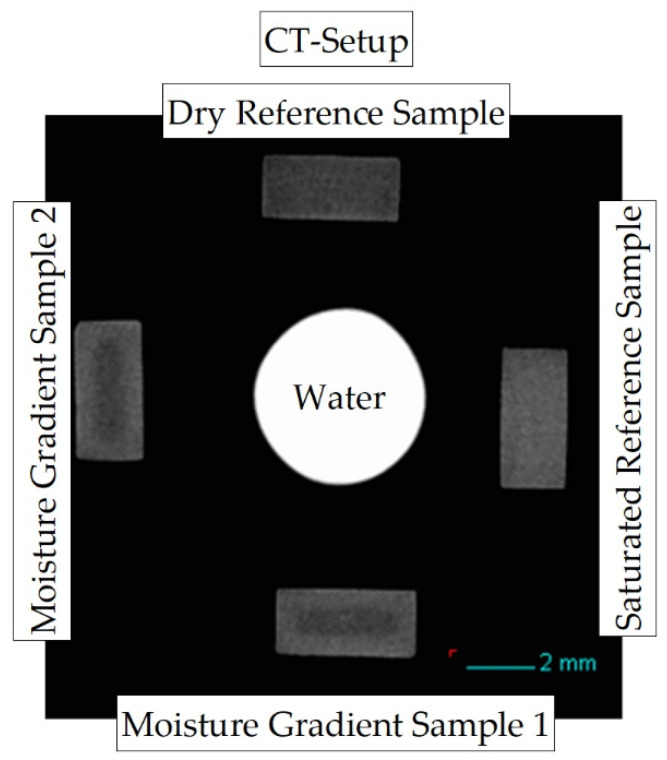
Reconstructed cross-section through the CT measurement setup to map concentration distributions due to X-ray absorption differences at low energy between PA6 and water.

The sample arrangement of the measurement setup is shown in Figure 2. During each measurement, five sample positions within the measurement setup are used: two samples with different concentration distributions and varying integral water contents (gradient samples 1 and 2), which can be seen on the positions “bottom” and “left” in the measurement setup shown. In addition, two reference samples are required. A dry sample (position “top”), which is (vacuum) dried and has an initial moisture content of <0.2%, and a completely water-saturated sample (position “right”) with a storage time of approx. 400 h in a water bath at room temperature and approx. 50 h at 80 °C water bath temperature. Both reference samples are required in the reconstruction to determine the mean absorbance of dry and fully water-saturated material, respectively. In addition, a capillary with water is measured in the middle of the samples as a further reference.

Based on the X-ray absorption coefficients obtained respectively, a water saturation W [%] can be calculated for each voxel using Equation (3), assuming a linear correlation between grey value and water content. This corresponds to the temperature- and geometry-dependent maximal possibility of the PA6 to absorb water, and is accordingly a normalised quantity. The water saturation is calculated as the percentage water saturation *W* [%] of the sample from its absorption $\mu_{Sample}\;\left[-\right]$ and relates this to the mean absorption of the dry reference ${\bar{\mu }}_{R0\%}\;\left[-\right]$ and the water-saturated reference ${\bar{\mu }}_{R100\%}\;\left[-\right]$ measured in the same measurement procedure.

(3) $$W=\frac{\mu_{Sample}-{\bar{\mu }}_{R0\%}}{{\bar{\mu }}_{R100\%}-{\bar{\mu }}_{R0\%}}\cdot 100\;\left[\%\right]$$

### 2.3. Determination of Concentration-Dependent Mutual Diffusion Coefficients

Diffusion coefficients can be derived from experimentally determined sorption curves with the help of Fick’s laws. A detailed description of the approach applied on the basis of Fick’s laws and the experimental determination of the concentration-dependent diffusion coefficients can be found in [16], hence only the basic formulas are provided here. The mass increase M(t) due to water absorption is calculated with the mass $m_{0}\;\left[\mathrm{kg}\right]$ of the dry specimen and the mass $m_{t}\;\left[\mathrm{kg}\right]$ considered at time t as shown in Equation (4):

(4) $$M\left(t\right)=\frac{m_{t}-m_{0}}{m_{0}}\cdot 100$$

Based on the equations in The Mathematics of Diffusion by Crank [28], a correlation between the mass change $M_{t}/M_{max}$ during the sorption process and the diffusion coefficient $D\;\left[m^{2}/s\right]$ can be derived according to Abacha [29]. In Equation (5), the assumption is considered that mass transport is exclusively one-dimensional and only dependent on the sample thickness d [mm].

(5) $$\frac{M_{t}}{M_{max}}=\frac{4}{d}\sqrt{\frac{Dt}{\pi }}$$

The assumptions made by Abacha [29] to simplify Equation (5) only consider water absorption via two sample surfaces. However, if cuboid samples are considered, the influence on sorption by the existing side surfaces of the sample is not negligible. The thicker the considered cuboid sample, the higher the influence of the side surfaces, so that instead of the sample thickness d, the volume-to-surface ratio (V/A ratio) is included in Equation (6) [22].

(6) $$\frac{M_{t}}{M_{max}}=\frac{2A_{tot\;}}{V}\cdot \sqrt{\frac{Dt}{\pi }}$$

For this purpose, the relationship in Equation (7) is transformed according to the diffusion coefficient $D\;\left({\bar{c}}_{\infty ,j}\right)$ and the V/A ratio of the respective geometry to be analysed is considered. The relationship shown in Equation (7) is used to determine the factor k, which is valid in the range of the linear sorption increase and corresponds to the slope of the curve.

(7) $$D\left({\bar{c}}_{\infty ,j}\right)=\frac{\pi }{16}\cdot {\left(\frac{\frac{M_{t}\left({\bar{c}}_{\infty ,j}\right)}{M_{max}\left({\bar{c}}_{\infty ,j}\right)}}{\frac{\sqrt{t}}{2\cdot \frac{V}{A_{tot}}}}\right)}^{2}=\frac{\pi }{16}\cdot k^{2}\;$$

In previous work, several approaches for determining the concentration-dependent diffusion coefficients have been considered and directly compared [30]. The most promising approach, by Inoue and Hoshino [21], will be examined. According to this, the diffusion coefficient D determined according to Equation (8) corresponds approximately to the integral of the mutual diffusion coefficient $\hat{D}$ over the respective saturation concentration ${\bar{c}}_{\infty }$ multiplied by the reciprocal of the saturation concentration ${\bar{c}}_{\infty }$, and the following Equation (5) becomes applicable:

(8) $$D\approx \;\frac{1}{{\bar{c}}_{\infty }}\;\int_{0}^{{\bar{c}}_{\infty }}\hat{D}d{\bar{c}}_{\infty }$$

The mutual diffusion coefficient $\hat{D}$ describes the process of directed diffusion along the concentration gradient and is determined by the concentration-dependent change of the product of the determined diffusion coefficient and its respective saturation concentration. Using the mutual diffusion coefficient $\hat{D}$, the authors achieve higher agreement between the model and measured data, hence this approach is subsequently applied in this study. In addition, this approach allows the calculation of a self-diffusion of the material in a completely dry state $\left({\bar{c}}_{\infty }=0\;\mathrm{ppm}\right)$. Therefore, the respective diffusion coefficient D is multiplied with its corresponding saturation concentration ${\bar{c}}_{\infty }$ and the self-diffusion of the material can be determined by extrapolation. The determination of the concentration-dependent diffusion coefficients from the experimental data and their conversion into mutual diffusion coefficients by using Formula (5) as well as the validation of the numerical calculation is described in detail in the work by Sambale et al., [11] and [30]. The mutual diffusion coefficients ${\hat{D}}_{80\;{}^{\circ}C}\left(c_{j}\right)$ determined there are listed in Table 2 and are subsequently applied for the numerical calculation of the sorption behaviour.

### 2.4. Finite Element Model Used for Mass Diffusion Analysis

For the numerical modelling of the sorption behaviour, the mass diffusion analysis provided in Abaqus from Dassault Systèmes (France) is used, which is based on the principles of Fick’s laws [31]. A detailed explanation of the numerical calculation can be found in [32,33] and is described in detail in [16] for the numerical approach of the presented FE calculation. In the following, the most important correlations of the sorption calculation from [16] are summarised using Equations (9)–(13). The mass diffusion analysis is performed considering the concentration of the diffusing substance c [ppm] and the concentration flux J [m⁄s] of the diffusing phase into a volume $V\;\left[m^{3}\right]$ with surface area $A\;\left[m^{2}\right]$ with the normal vector n [−] perpendicular to the surface in Equation (9). The term n·J describes the concentration flow through the surface A.

(9) $$\int_{V}^{\;}\frac{dc}{dt}dV+\int_{A}^{\;}n\cdot J\;dA=0$$

Using Gauss’s integral theorem, Equation (9) can be transformed into a volume integral so that Equation (10) is valid.

(10) $$\int_{V}^{\;}\left(\frac{dc}{dt}+\frac{\partial }{\partial x}\cdot J\right)dV=0$$

To calculate the concentration, a solubility variable ϕ [ppm] is defined, which is referred to as the activity of the diffusing material and describes the degrees of freedom of the network nodes. It is calculated by means of Equation (11) from the mass concentration c [ppm] of a substance and its solubility s [−] in the material of interest. The solubility variable ϕ, as a sort of normalised concentration, enables a continuous calculation of the solubility across the interfaces of different materials as well.

(11) $$\phi =\frac{c}{s}$$

Using the definition of the solubility variable, the concentration flux J can be calculated from Fick’s first law, resulting in Equation (12), shown for calculating the concentration flux.

(12) $$J=-D\cdot \left(s\frac{\partial \phi }{\partial x}+\phi \frac{\partial s}{\partial x}\right)$$

If the simplification is adopted so that only homogeneous base material is considered, the solubility s is to be regarded as constant and Equation (12) can be summarised as shown in Equation (13).

(13) $$J=-s\cdot D\frac{\partial \phi }{\partial x}$$

## 3. Results and Discussion

### 3.1. CT Measurements and Reconstruction and FE Analysis for Water Sorption with Experimentally Determined Diffusion Coefficients

Figure 2 shows the reconstructed cross-section of three samples of the dimensions (4 × 2 × 80) mm^3^, selected from the center of the sample, aged for different periods in the water bath at room temperature as a greyscale image. The variation in the storage periods in water results in different integral water contents for the samples determined by mass increase, which are given as integer values.

**Figure 3:**
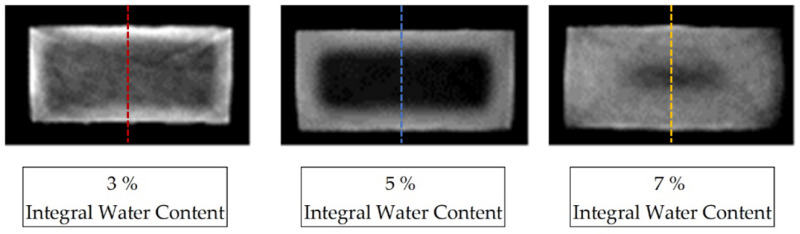
CT reconstruction of three PA6 samples with concentration distributions at different integral water contents, saturated at 23 °C in a water bath. The images have been magnified and are contrast-spread.

**Figure 4:**
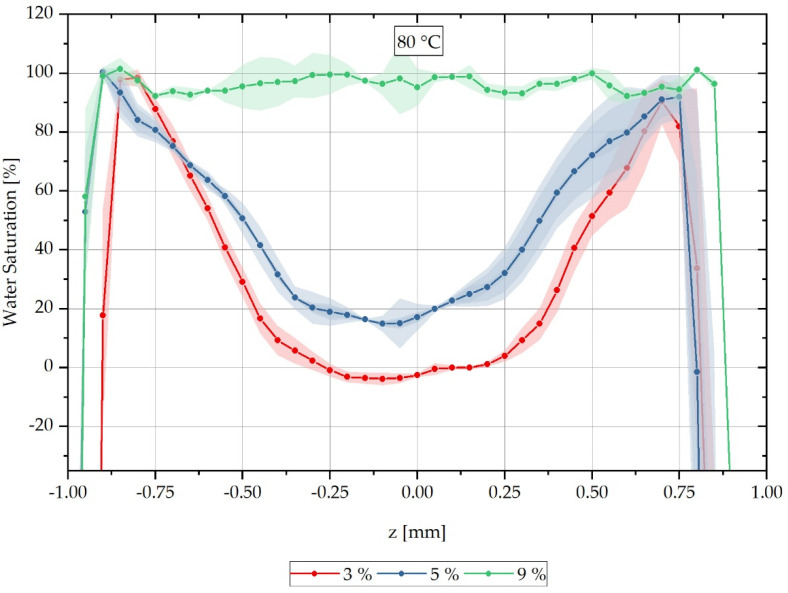
Spatially resolved distribution of water concentration reconstructed from CT data across the sample thickness at different integral water contents during sorption process in a water bath at 80 °C.

For each sample, a characteristic grey value distribution over the sample cross-section can be recognised, which is evaluated subsequently along the dotted lines inserted in the samples shown in Figure 3, and which correspond to the reconstruction method of the water concentration distribution described in Equation (3). The dotted lines in Figure 3 represent the z-direction in the sample coordinate system. Figure 4 shows the local water saturation along the samples’ z-direction for the integral water contents of 3% (red line), 5% (blue line) and 9% (green line) for the water bath temperature of 80 °C. The water bath temperature of 80 °C was initially chosen to be considerably above the glass transition range $T_{g}$ of approx. 60 °C of dry PA6.

The location-dependent saturation profiles shown are measured on three samples each and are then arithmetically averaged so that a standard deviation can be given for each measured value in the form of an error tube.

Figure 4 shows the relative concentration curve reconstructed from the CT measurements over the cross-section of the respective measured samples at a water bath temperature of 80 °C. The samples with an integral water content of 3% are shown with a red-dotted line and those with 5% in blue. The samples shown with a green-dotted line are samples with an integral water content of 9%. It can be seen that the water concentration distribution changes significantly for the different integral water contents.

Furthermore, it can be seen that for the integral water content of 3% for both water bath temperatures, negative water saturations are also determined in the reconstruction. These negative values occur when a higher average X-ray absorption ${\bar{\mu }}_{R0\%}$ is measured for the completely dry reference sample than the simultaneously measured gradient sample has in its dry core as a locally determined absorption value $\mu_{Sample}$. For this purpose, negative reconstruction values are shifted to the water saturation of 0% on the ordinate in the following, so that a comparison of the CT measurements with the FE calculations can be accomplished. The applied shift parameters are listed in Table 3 and used according to Equation (14). Here the value $y_{shift}$ results from the multiplication of the shift factor $a_{y}$ with the original measured value $y_{0}$ and the subsequent addition of the factor $b_{y}$.

(14) $$y_{shift}=(a_{y}\cdot y_{0})+b_{y}$$

In addition, it can be seen that in the boundary layer of approx. ±1.0 to ±0.8 mm a clearly too-low water content is assumed, although a complete saturation of the boundary layer can be assumed during sorption in a water bath. This insufficient water content can be attributed to two different effects: First, the density gap between air and material is smeared over two voxels so that, due to the geometric measurement arrangement, an area of approximately 110 µm (with a voxel size of 55 µm) is reconstructed with insufficient water content. For this reason, an examination of the water-induced swelling on the basis of the measured CT data is not possible due to the large voxel size and will not be considered further. The required scan time of 1.5 h is sufficiently large for re-drying effects in the water-saturated sample boundary to show a relevant influence, since the surrounding air in the laboratory standard climate (23 °C/50% r.H.) has a lower moisture content than the sample boundary layer. However, re-drying is a continuously changing effect on the moisture content of the sample boundary layer during the scan time, which cannot be sensibly determined with this measurement setup. For the above reasons, the measured maximum values are assumed to show a complete water saturation of 100% at the sample boundary and the shift parameters used for the abscissa are given in Table 4 and used according to Equation (15). The factors used in Formula (15) are to be seen correspondingly to the factors used in Formula (14) in the z-direction. This enables a direct comparison of the measurement results with the FE calculation model for all samples measured by CT.

(15) $$z_{shift}=(a_{z}\cdot z_{0})+b_{z}$$

For the FE calculation of the concentration distribution within the sample cross-section, the sorption calculation described in Section 2.4 based on the mass diffusion analysis in Abaqus is used. The concentration-dependent mutual diffusion coefficients are used according to Table 2 for the values given at a water bath temperature of 80 °C. The analysed sample geometry corresponds to the bar-shaped sample of dimensions (4 × 2 × 80) mm^3^ used in the CT scans. Within a Python script, the concentration is normalised to the solubility s (see Equation (11)) from the FE calculation is evaluated as a function of the selected spatial coordinate z and additionally normalised to the respective maximum value of the saturation $c_{\infty }\left(T\right)$, which depends on the water bath temperature, so that it corresponds to the water saturation W in percent.

**Figure 5:**
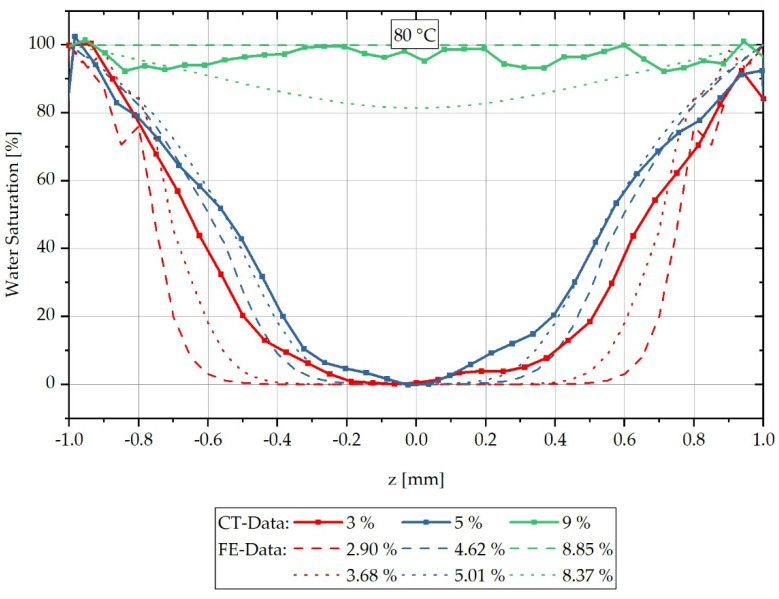
Comparison of the concentration distribution over sample thickness measured by CT and the calculated concentration distribution during sorption in a water bath at 80 °C.

The reconstructed results of the CT measurement method, shifted according to Equations (14) and (15) and Table 3 and Table 4, are compared with the calculated concentration distributions in Figure 4 for a water bath temperature of 80 °C. Since both the concentration distributions and the integral water contents are determined from averages of the values of several samples, the calculated concentration distributions are shown for the time step directly below and directly above the integral water contents of the experimentally determined data. The error tubes shown in Figure 4 are omitted in the comparison with the FE analysis in Figure 5 for readability.

Figure 5 shows the water saturation distribution of the reconstructed CT measurements combined with the concentration distribution calculated by FEM over the sample thickness for a water bath temperature of 80 °C. The measured integral water contents of 3%, 5% and 9% are compared with the calculated curves of the time steps directly below or above, which result in integral water contents directly below or above the measured values considered. For the values shown in red, which belong to 3% integral water content, it can be seen that the calculated concentration gradient tends to slope too steeply in comparison to the measured gradient, so that the calculation initially slightly overestimates the concentration gradient for low water contents at high moisture gradients. For the values shown in blue for an integral water content of 5%, the calculated concentration courses are in good approximation with the reconstructed CT measurement data and in particular the gradient change in the water distribution curve is reproduced qualitatively very well by the FE model. For the reconstructed values of the samples with 9% integral water content, a complete water saturation and thus constant concentration distribution can be recognised, which is reproduced by the FE calculation with the higher integral water content of 8.85%. The calculation with a lower integral water content of 8.37% predicts that, according to the calculation, there is no complete water saturation yet at this time step. However, both calculated concentration distributions have a lower water content than the integral 9% water content assumed in the measurement. The lower integral water contents result from the experimentally determined, temperature-dependent maximum saturation of the material, which corresponds to a saturation of 8.9% for a water bath temperature of 80 °C. The stated 9% water content of the CT reconstruction results from the mean value of the measured samples rounded to integer values. Overall, however, it can be summarised that the FE calculation of the concentration distribution corresponds in a good approximation to the reconstructed concentration distribution from CT measurements, and thus validates it.

### 3.2. CT Measurements and Reconstruction and FE Analysis for Water Sorption with Diffusion Coefficients Determined Using a Factor Comparison

**Figure 6:**
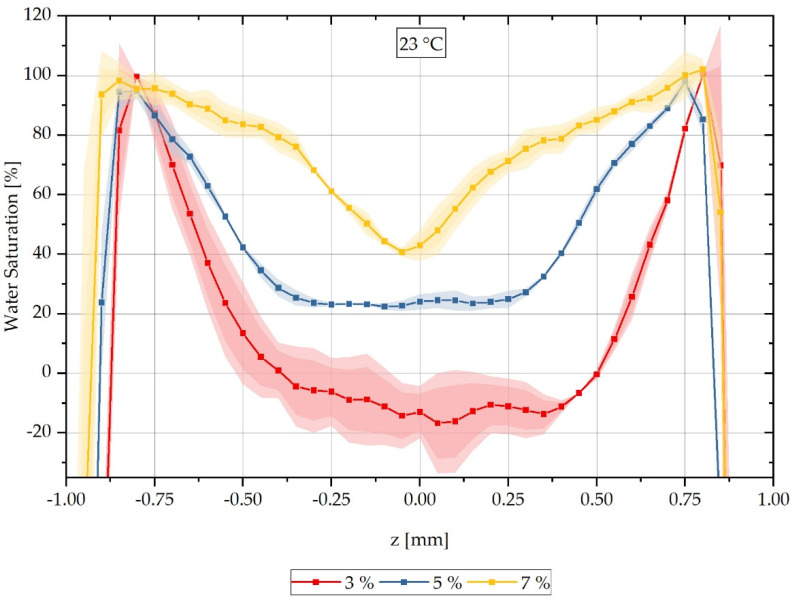
Spatially resolved distribution of the water concentration over the sample thickness reconstructed from the CT data as at different integral water contents during sorption in the water bath at 23 °C.

In addition to assessing the water distribution at different times over the cross-section of samples saturated at a water bath temperature of 80 °C, the variation in the water bath temperature is also of interest. Here, water saturation at a room temperature of 23 °C is considered in order to be able to evaluate diffusion processes and concentration distributions at room temperature. Similar to the previous series of measurements at a water saturation of 80 °C, CT measurements are conducted on samples with different integral water contents after storage in a water bath at 23 °C. The results of the CT measurements are presented as a reconstructed relative concentration distribution over the cross-section of the respective measured samples in Figure 6. Samples with an integral water content of 3% are shown with a red-dotted line, and those with 5% in blue. For a water bath temperature of 23 °C, in contrast to the previously shown results at 80 °C, measurements are also carried out on samples with an integral water content of 7% instead of 9%; these are shown with a yellow-dotted line. Here, it was decided to select 7% integral water content, since a pronounced moisture gradient is to be expected due to the lower water content and thus not an (almost) constant water distribution as in the previously analysed 9% integral water content. It can also be seen for this measurement series that the concentration distribution for the different integral water contents differ significantly from each other and the higher the integral water content of the samples, the lower the concentration gradients. For the two integral water contents of 3% and 5%, respectively, constant values can be seen in the area of the sample centre; this cannot be seen for the integral water content of 7%.

**Figure 7:**
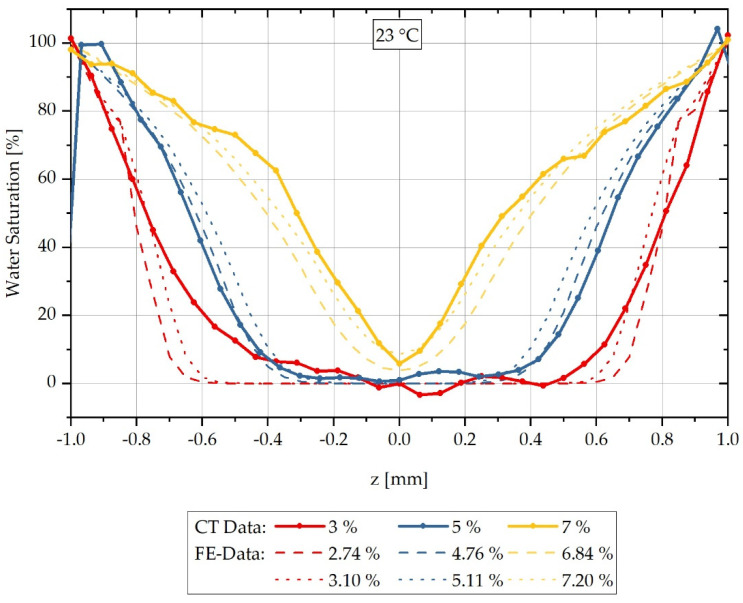
Comparison of the concentration distribution measured by CT and the calculated distribution over the sample thickness during sorption in a water bath at 23 °C.

Analogously to Figure 4, it can be seen that the water concentration distribution changes significantly for the different integral water contents and a delay of the water saturation to negative values occurs both in the boundary area of the samples and in the middle area of the concentration curves. Based on the previously mentioned reasons in Section 3.1., these can be shifted to a maximum saturation of 100% water saturation in the boundary region and to 0% saturation in the sample core of the 3% and 5% samples, respectively, by using shift factors. The shift factors are listed in Table 5. They are applied similarly to the 80 °C water bath temperature results using Equations (14) and (15) and the reconstruction results thus obtained are shown in direct comparison with the calculated concentration curves in Figure 7.

For the FE calculation of the concentration curves over the sample cross-section, it should be noted that the concentration-dependent diffusion coefficients previously used for the simulation originate from experimentally measured sorption curves of a temperature of 80 °C. The experimental procedure for determining concentration-dependent diffusion coefficients as well as the maximum saturation is time-consuming due to the required sorption times and becomes even more tedious for lower water bath temperatures and lower surrounding water concentrations due to the reduction in the diffusion rate at lower temperatures. A conversion of the diffusion coefficients with the help of the Arrhenius approach is also not possible, as the range of validity of the formula is only given below the glass transition region $T_{g}$ [34]. The approach presented by Vrentas et al. [35] for calculating the diffusivity of water based on the free-volume theory with a range of validity above $T_{g}$ cannot be used for conversion in this case either. PA6, however, has a $T_{g}$ of approx. 60 °C in the dry state but shifts it to −20 °C for completely water-saturated material [10,11]. Although the chosen 23 °C of the water bath temperature is below the glass transition for dry material, a $T_{g}$ shift is induced locally by the water absorption [11]. Since both models mentioned are only valid above or below, but not within the glass transition, an alternative approach for determining concentration-dependent diffusion coefficients is developed.

For the approach used, only the diffusion coefficient for saturation of the samples in a water bath at 23 °C is determined experimentally, since in this case sample saturation is still reached within a moderate period of approx. 400 h (≙ 16 days) for the present sample geometry. This is determined from the measured sorption curve using Equation (7) and is $D_{23{}^{\circ}C,\;Water}=3.6\times 10^{-13}m²/s$. However, in the FE calculations performed, the diffusion coefficients $\hat{D}\left(c_{j}\right)$ converted using Equation (8) are used according to the approach of Inoue et al. [21], which is based on the principle of mutual diffusion, and are converted from the diffusion coefficients previously determined experimentally at different ambient concentrations. So that FE analyses can still be calculated for saturation at 23 °C with concentration-dependent diffusion coefficients, the correlations between the different concentration-dependent mutual diffusion coefficients must be determined. For this purpose, factors $K\left(c_{j}\right)$ are determined, which are calculated from the ratio of the respective concentration-dependent, mutual diffusion coefficient $\hat{D}\left(c_{80{}^{\circ}C,\;Water}\right)$ to the experimentally determined diffusion coefficient for the water bath saturation at 80 °C according to Equation (16).

(16) $$K\left(c_{j}\right)=\frac{{\hat{D}}_{80{}^{\circ}C}\left(c_{j}\right)}{D_{80{}^{\circ}C}\left(c_{,\;Water}\right)}$$

The factors $K\left(c_{j}\right)$ are subsequently multiplied according to Equation (17) with the experimentally determined diffusion coefficient for the saturation in the water bath at 23 °C ${\hat{D}}_{23\;{}^{\circ}C}\left(c_{Water}\right)$ so that the respective concentration-dependent mutual diffusion coefficients ${\hat{D}}_{23{}^{\circ}C}\left(c_{j}\right)$ result and the values are listed in Table 6.

(17) $${\hat{D}}_{23{}^{\circ}C}\left(c_{j}\right)=K\left(c_{j}\right)\cdot {\hat{D}}_{23{}^{\circ}C}\left(c_{Water}\right)$$

By using the concentration-dependent values for the mutual diffusion coefficients ${\hat{D}}_{23{}^{\circ}C}\left(c_{j}\right)$ listed in Table 6, the concentration curves for the different integral water contents can be calculated by FE. These are shown in Figure 7 in comparison with the reconstructed CT measurement data shifted by means of Table 5. For reasons of readability, the error tubes of the concentration curves averaged from three measurements each are omitted.

The results of the calculated and measured concentration distributions shown in Figure 7 indicate that for sorption in a water bath at 23 °C the FE calculation also agrees with the CT measurements in a good approximation, but in principle slightly overestimates the concentration gradient. For each measured integral water content, a calculated integral water content below and above the measurement is shown corresponding to Figure 7.

The different concentration gradients from the maximum saturation at the boundaries to the concentration minimum at the centre of the samples are simulated by the FE model for all measurements considered. With an integral water content of 3%, only the constant, dry region within the sample is overestimated, so that a discrepancy occurs here. The results presented in Figure 7 show that the conversion by factors is a reasonable method to determine concentration-dependent, mutual diffusion coefficients from only one experimentally determined diffusion coefficient at high surrounding concentration. However, a requirement for this is that concentration-dependent, mutual diffusion coefficients have previously been determined for a different surrounding temperature.

The approach described in Section 2.2. shows that low-energy CT measurements can be used to represent concentration distributions due to sorption processes in PA6. In addition, it can be stated that the FE calculation method introduced in Section 2.3. based on the mass diffusion analysis provided in Abaqus can resolve the concentration distribution over the sample cross-section in a good approximation. Concentration-dependent, mutual diffusion coefficients are used for the FE calculation. In conclusion, concentration distributions of water in PA6 can be calculated with the developed FE method with sufficient accuracy.

## 4. Conclusions

In the present work, a method for the characterisation and numerical computation by means of FEM of water concentration distributions during the sorption process of PA6 is developed. For this purpose, a possibility is first shown using low-energy CT scans, which allows concentration distributions in PA6 to be represented and reconstructed based on the X-ray absorption differences between water and PA6. This allows a spatially resolved quantification of the water content over a sample cross-section. Scans at different times of the sorption process also allow the concentration change to be observed over time. The reconstruction of different concentration distributions at different times allows these to be compared with a numerical calculation method developed previously. This FE calculation is based on the mass diffusion analysis in Abaqus and uses concentration-dependent, mutual diffusion coefficients that consider the present V/A ratio. Due to known inaccuracies of the reconstruction method in the area of quantification as well as the determination of the moisture in the sample boundary, the reconstructed concentration distributions can be shifted by applying shift factors. The comparison of the numerically calculated and the reconstructed concentration distributions shows that the FE calculation tends to underestimate the concentration distribution over the sample cross-section, especially in the case of low integral water contents, but generally reproduces it in a very good approximation, so that the FE calculation can be regarded as experimentally validated.

In a further step, the water bath temperature used for conditioning is reduced from 80 °C to 23 °C. For a numerical calculation, the diffusion coefficients used, which are dependent on the temperature and measured at 80 °C, must therefore be converted to the altered temperature of 23 °C. Due to the present glass transition temperature $T_{g}$ for dry PA6 of about 60 °C, the sorption from temperatures above $T_{g}$ to temperatures below the glass transition range is also altered by the change in the water bath temperature, so that the usual conversion methods such as the Arrhenius approach and the free-volume theory for a temperature change in diffusion coefficients cannot be used. An experimental determination of the concentration-dependent diffusion coefficients is rather time-consuming, especially for the required low water concentrations, so only a diffusion coefficient for a water bath storage at 23 °C can be determined. In order to obtain concentration-dependent, mutual diffusion coefficients for 23 °C from this, a factor comparison is presented in the present work. It is determined from the concentration-dependent, mutual diffusion coefficients for 80 °C as the quotient of the respective nearest lower value and the currently considered diffusion coefficient and multiplied by the diffusion coefficient of the lower water bath temperature. This results in the diffusion coefficient for the respective lower concentration. The thus-calculated concentration-dependent mutual diffusion coefficients for 23 °C are applied for the numerical calculation of the concentration distribution of the water at different integral water contents and compared with reconstructed CT measurements of the corresponding states. It is shown that the concentration distributions calculated numerically agree very well with the reconstruction although the diffusion coefficients used are recalculated to lower water bath temperatures.

In further work, an optimisation of the CT measurement setup should be considered, as the resulting voxel size of 55 µm with two voxels smearing due to the existing X-ray absorption difference between air and material causes an inaccuracy in the boundary region of the sample being too high. A reduction in the voxel size with this inaccuracy in the boundary area would lead to the possibility of a detailed observation of re-drying effects and, in addition, water-induced swelling could possibly also be observed with sufficient resolution. In addition, further water bath temperatures for water sorption could be considered so that the presented factor comparison could be further validated. In addition to the concentration distribution due to water sorption, concentration distributions due to desorption effects could also be both experimentally characterised and numerically calculated.

## Figures and Tables

**Table 1:** Measurement parameters of the low-energy CT system used to determine water concentration distributions in PA6.

Measurement Parameters	Parameter Values
X-ray tube voltage	25 kV
Anode current	0.8 mA
Prefilter	1 mm aluminum
Voxel size	55 µm
Scan time	1.5 h

**Table 2:** Experimentally determined concentration-dependent diffusion coefficients and resulting calculated reciprocal diffusion coefficients from Sambale et al. [16].

Water Concentration	Diffusion Coefficient at 80 °C	Mutual Diffusion Coefficient at 80 °C
c	$D_{80\;{}^{\circ}C}\left(c_{j}\right)$	${\hat{D}}_{80\;{}^{\circ}C}\left(c_{j}\right)$
[ppm]	[m²/s]	[m²/s]
0	−	$2.5\cdot 10^{-12}$
30,000	$7.2\cdot 10^{-11}$	$7.3\cdot 10^{-12}$
65,000	$1.0\cdot 10^{-11}$	$2.4\cdot 10^{-11}$
89,000	$1.9\cdot 10^{-11}$	$5.5\cdot 10^{-11}$

**Table 3:** Shift factors for the CT reconstruction in the ordinate direction to compensate for the grey values for completely dry material assumed to be too low for a water bath temperature of 80 °C.

Water Bath Temperature 80 °C	Integral Water Content		
	3%	5%	9%
$a_{y}$	1.25	1.20	1.15
$b_{y}$	0.05	0.08	0.02

**Table 4:** Shift factors for the CT reconstruction in the abscissa direction to compensate for the grey values for completely dry material assumed to be too low for a water bath temperature of 80 °C.

Water Bath Temperature 80 °C	Integral Water Content		
	3%	5%	9%
$a_{z}$	0.98	1.20	1.00
$b_{z}$	+4	−15	0

**Table 5:** Shift factors for the CT reconstruction in the ordinate direction ($a_{y}$ and $b_{y}$) to correct the grey values assumed to be too low for completely dry material and shift factors in the abscissa direction ($a_{z}$ and $b_{z}$) to correct the boundary resolution as well as the re-drying effects during the measurement time for a water bath temperature of 23 °C.

Water Bath Temperature 23 °C	Integral Water Content		
	3%	5%	7%
$a_{y}$	1.25	1.21	1.25
$b_{y}$	0	0.05	0.05
$a_{z}$	0.90	1.37	1.60
$b_{z}$	+13	−22	−37

**Table 6:** Determination of the concentration-dependent mutual diffusion coefficients from an experimentally determined diffusion coefficient for a temperature of 23 °C by factor comparisons from the experimentally determined values converted into mutual diffusion coefficients for different ambient concentrations at 80 °C.

Water Concentration	Diffusion Coefficient at 80 °C	Mutual Diffusion Coefficient at 80 °C	Factor	Diffusion Coefficient at 23 °C	Mutual Diffusion Coefficient at 23 °C
c	$D_{80{}^{\circ}C}\left(c_{j}\right)$	${\hat{D}}_{80{}^{\circ}C}\left(c_{j}\right)$	$K\left(c_{j}\right)$	$D_{23{}^{\circ}C}\left(c_{Water}\right)$	${\hat{D}}_{23{}^{\circ}C}\left(c_{j}\right)$
[ppm]	[m²/s]	[m²/s]	[−]	[m²/s]	[m²/s]
0	−	$2.5\cdot 10^{-12}$	0.14	−	$5.1\cdot 10^{-14}$
30,000	$7.2\cdot 10^{-11}$	$7.3\cdot 10^{-12}$	0.39	−	$1.5\cdot 10^{-13}$
65,000	$1.0\cdot 10^{-11}$	$2.4\cdot 10^{-11}$	1.30	−	$4.7\cdot 10^{-13}$
89,000	$1.9\cdot 10^{-11}$	$5.5\cdot 10^{-11}$	2.96	$3.6\cdot 10^{-13}$	$1.1\cdot 10^{-12}$
